# Rotavirus.

**DOI:** 10.3201/eid0404.980406

**Published:** 1998

**Authors:** U D Parashar, J S Bresee, J R Gentsch, R I Glass

**Affiliations:** Viral Gastroenteritis Section, Centers for Disease Control and Prevention, Atlanta, GA 30333, USA. uap2@cdc.gov

## Abstract

Rotavirus, the most common diarrheal pathogen in children worldwide, causes approximately one third of diarrhea-associated hospitalizations and 800,000 deaths per year. Because natural infection reduces the incidence and severity of subsequent episodes, rotavirus diarrhea might be controlled through vaccination. Serotypespecific immunity may play a role in protection from disease. Tetravalent rhesus-human reassortant rotavirus vaccine (RRV-TV) (which contains a rhesus rotavirus with serotype G3 specificity and reassortant rhesus-human rotaviruses with G1, G2, and G4 specificity) provides coverage against the four common serotypes of human rotavirus. In clinical trials in industrialized countries, RRV-TV conferred 49% to 68% protection against any rotavirus diarrhea and 61% to 100% protection against severe disease. This vaccine was licensed by the U.S. Food and Drug Administration on August 31, 1998, and should be cost-effective in reducing diarrheal diseases in industrialized countries. The vaccine's efficacy and cost-effectiveness in developing countries should be evaluated.


### Rotavirus

**Umesh D. Parashar, Joseph S. Bresee,
Jon R. Gentsch, and Roger I. Glass**
Centers for Disease Control and Prevention, Atlanta, Georgia, USA

Rotavirus, the most common diarrheal pathogen in children worldwide, causes approximately one third of diarrhea-associated hospitalizations and 800,000 deaths per year. Because natural infection reduces the incidence and severity of subsequent episodes, rotavirus diarrhea might be controlled through vaccination. Serotype-specific immunity may play a role in protection from disease. Tetravalent rhesus-human reassortant rotavirus vaccine (RRV-TV) (which contains a rhesus rotavirus with serotype G3 specificity and reassortant rhesus-human rotaviruses with G1, G2, and G4 specificity) provides coverage against the four common serotypes of human rotavirus. In clinical trials in industrialized countries, RRV-TV conferred 49% to 68% protection against any rotavirus diarrhea and 61% to 100% protection against severe disease. This vaccine was licensed by the U.S. Food and Drug Administration on August 31, 1998, and should be cost-effective in reducing diarrheal diseases in industrialized countries. The vaccine's efficacy and cost-effectiveness in developing countries should be evaluated.

In 1973, Bishop and colleagues observed by electron microscopy, in the duodenal epithelium of children with diarrhea, a 70-nm virus, subsequently designated rotavirus (Latin, *rota* = wheel) because of its appearance (Figure 1) (1). Before this discovery, a bacterial, viral, or parasitic etiologic agent could be detected in only 10% to 30% of children with diarrhea. Within 5 years, rotavirus was recognized as the most common cause of diarrhea in infants and young children worldwide, accounting for approximately one third of cases of severe diarrhea requiring hospitalization (2-4). In the United States, approximately 2.7 million children <5 years of age are affected by rotavirus diarrhea each year, resulting in 500,000 physician visits and 50,000 hospitalizations at an estimated $274 million in medical care and more than $1 billion in societal costs (5,6). The cost of rotavirus diarrhea is even higher in developing countries, where 20% to 70% of hospitalizations and 800,000 of the three million deaths per year from diarrhea are caused by this pathogen (Figure 2) (7,8). Recognition of rotavirus as a major cause of diarrhea in children led to extensive research for interventions to reduce the incidence of this disease.

Address for correspondence: Umesh D. Parashar, Viral Gastroenteritis Section, Mail Stop G04, Centers for Disease Control and Prevention, 1600 Clifton Road, NE, Atlanta, GA 30333; fax: 404-639-3645; e-mail: uap2@cdc.gov.

Because rotavirus infects virtually all children ≤ 3 to 5 years of age in both industrialized and developing countries, improving water, food, and sanitation appeared unlikely to reduce disease incidence. Instead, early studies identified epidemiologic features indicating that rotaviral disease might best be

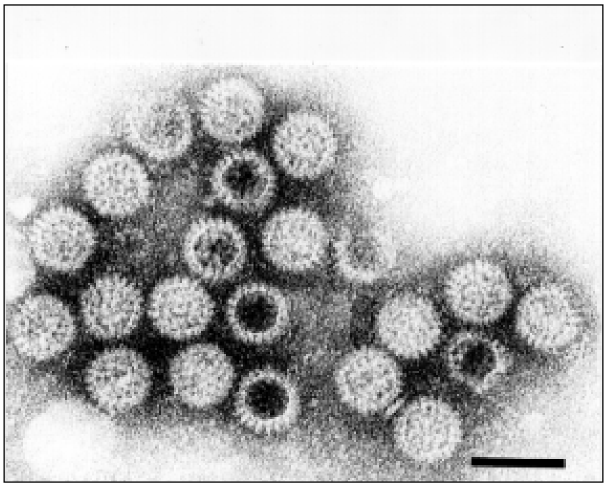

Figure 1. Rotavirus particles visualized by immune electron microscopy in stool filtrate from child with acute gastroenteritis. 70-nm particles possess distinctive double-shelled outer capsid. Bar = 100 nm.

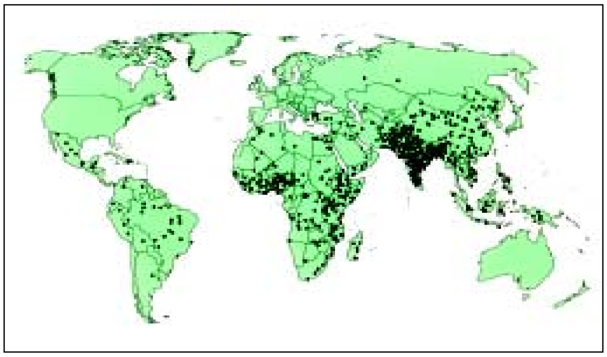

Figure 2. Estimated global distribution of the 800,000 annual deaths caused by rotavirus diarrhea. Reprinted with permission from (8).

controlled through vaccination. Natural immunity was suggested by the infrequent occurrence of more than one episode of rotavirus diarrhea in a child and decreased incidence of disease with increasing age (9). Furthermore, protection increased with each new infection and was greatest against moderate to severe disease, less against mild illness, and least against asymptomatic infection (10). These findings implied that an attenuated rotavirus vaccine that simulates natural infection could induce protective immunity and that more than one dose of vaccine may be required.

Several rotavirus vaccine candidates developed in the past 3 decades have proven safe and effective in clinical trials. On August 31, 1998, only 25 years after the discovery of rotavirus, a live, oral, tetravalent rhesus-human reassortant rotavirus vaccine (RRV-TV) was licensed by the U.S. Food and Drug Administration, and this vaccine may soon be available for immunization of children. This paper reviews the biologic and epidemiologic characteristics of rotavirus, discusses the development of rotavirus vaccines, and identifies research needs for expediting the introduction of rotavirus vaccines into childhood immunization programs.

### Rotavirus Characteristics

### Morphology and Classification

Rotavirus, an icosahedral virus in the family *Reoviridae*, has a distinct morphologic appearance by negative-stain electron microscopy (3). The viral capsid is triple-layered; the inner layer (core) contains the virus genome, which comprises 11 segments of double-stranded RNA, each coding for products that are either structural viral proteins (VP) or nonstructural proteins (NSP) (Figure 3). The segmented genome of rotavirus readily reassorts during coinfection, a property that has been used in

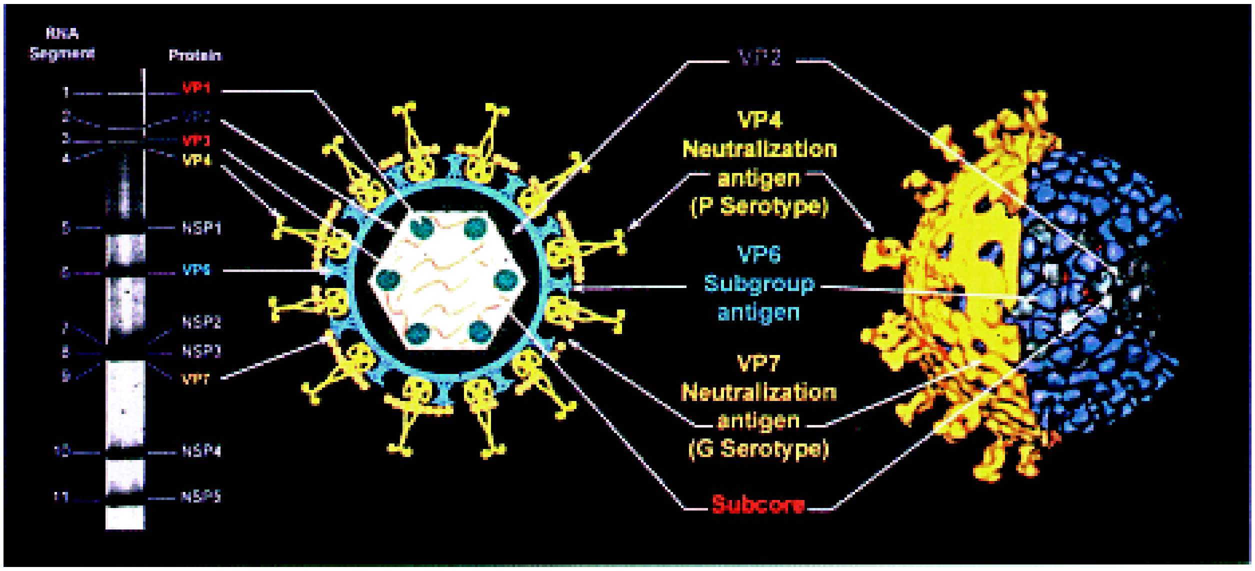

Figure 3. Gene coding assignments and three-dimensional structure of rotavirus particles. Double-stranded RNA segments separated on polyacrylamide gel (left) code for individual proteins, which are localized in the schematic of virus particle (center) or in different protein shells of virus (right). Outer capsid proteins VP4 and VP7 are neutralization antigens, which induce neutralizing antibody; protein that makes up intermediate protein shell, VP6, is the subgroup antigen. Reprinted with permission from (4).

developing vaccines and undoubtedly plays a role in virus evolution.

The major antigenic properties of rotaviruses—group, subgroup, and serotype—are determined by the viral capsid proteins. Rotavirus has seven major groups (A-G); most human strains belong to group A, although groups B and C have occasionally been associated with human illness. The product of the 6th gene of group A rotaviruses encodes VP6, the most abundant viral protein, which is the major determinant of group reactivity, the target of common diagnostic assays, and contains the antigen used to further classify rotaviruses into subgroups I and II. The outer capsid proteins, VP7, the glycoprotein or G-protein (encoded by gene 7, 8, or 9, depending on the strain), and VP4, the protease-cleaved or P-protein (encoded by gene segment 4), determine the serotype specificity and form the basis of the binary classification (G and P type) of rotaviruses. Both G and P proteins induce neutralizing antibodies and may be involved in protective immunity.

### Global Distribution of Rotavirus Strains

Fourteen G serotypes of rotavirus, 10 of which occur in humans, have been defined by cross-neutralization studies with polyclonal animal serum samples; these serotypes correlate with antigenic specificities of the VP7 glycoprotein. The characterization of P serotypes has been difficult because adequate reagents are not available. Eight P serotypes of human rotaviruses have been characterized. Additional VP4 gene variants have been identified, so ultimately the number of P serotypes may exceed 20. Theoretically, 80 different strains of rotavirus could result from various combinations of the known 10 G and 8 P serotypes of human rotaviruses. For vaccine development purposes, it is fortunate that only four common strains (P[8]G1; P[8]G3; P[8]G4; and P[4]G2) of rotavirus predominate globally (Figure 4) (11). However, the prevalence of rotavirus strains varies considerably from one geographic area to another, and unusual strains are common in several developing countries (e.g., unusual P[6] strains, including those with serotype G9 specificity, accounted for 9.5% of all rotaviruses from a multicenter collection in India) (12).

### Epidemiology

Rotaviruses are ubiquitous; 95% of children worldwide are infected by 3 to 5 years of age. The

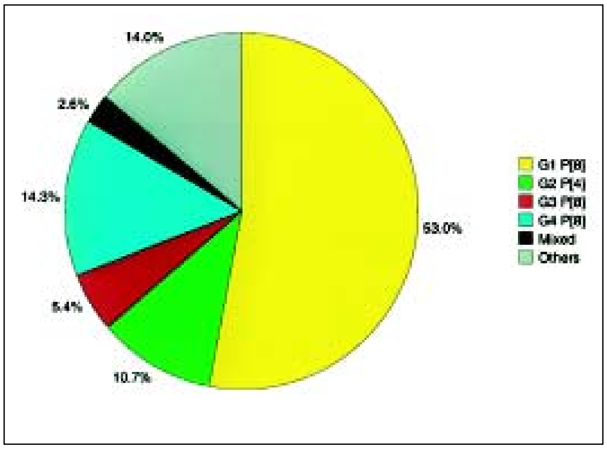

Figure 4. Distribution of rotavirus strains from a global collection of 2,748 strains. "Others" includes strains that were not typable. Adapted from (11).

prevalence of rotavirus infection in neonates has not been systematically examined, but high infection rates were documented in newborns in six hospitals in India (13). These infections, frequently asymptomatic, were caused by unusual strains of rotavirus. The incidence of clinical illness peaks among children ages 4 to 36 months, who are also at greatest risk for severe disease requiring hospitalization. Rotavirus infections of adults are usually subclinical but occasionally cause illness in parents of children with rotavirus diarrhea, immunocompromised patients (including those with HIV), the elderly, and travelers to developing countries. In temperate climates, rotavirus diarrhea occurs predominantly during the fall and winter; in tropical settings and in developing countries, seasonality is less marked (14). Peak rotavirus activity in the United States begins in the Southwest in autumn (October through December) and ends in the Northeast during spring (March through May) (Figure 5) (15); the reason for this pattern is unknown.

Rotaviruses are shed in large numbers during episodes of diarrhea, and usually are detectable by antigen enzyme immunoassays (EIA) up to 1 week after infection or for more than 30 days in immunocompromised patients. A recent study showed that as many as 30% of immunocompetent infants with severe rotavirus diarrhea may have virus detectable by polymerase chain reaction (PCR) for more than 25 days after hospital admission. The predominant

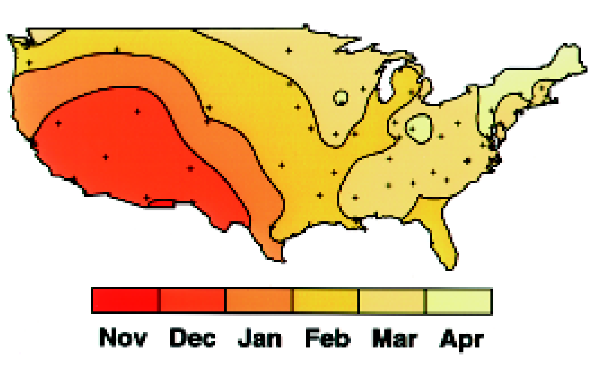

Figure 5. Average time of peak rotavirus activity in the contiguous 48 states, United States, July 1991 to June 1997. This contour plot was derived using the median value for time of peak activity for each laboratory. The surveillance system and analytic methods used to create this map are described in greater detail in (15). + indicates location of laboratories participating in the surveillance system.

mode of rotavirus transmission is fecal-oral. Spread through respiratory secretions, person-to-person contact, or contaminated environmental surfaces has also been speculated because of the high rates of infection in the first 3 years of life regardless of sanitary conditions, the failure to document fecal-oral transmission in several outbreaks of rotavirus diarrhea, and the dramatic spread of rotavirus over large geographic areas in the winter. Animal-to-human transmission does not appear to be common, although human rotavirus strains that possess a high degree of genetic homology with animal strains have been identified (16). Animal strains of rotavirus differ from those that infect humans.

### Pathogenesis and Immunity

Rotaviruses infect the mature absorptive villous epithelium of the upper two thirds of the small intestine. After replication in the upper small intestine, infectious particles are released into the intestinal lumen and undergo further replication in the distal areas of the small intestine. Infection is generally confined to the intestinal mucosa. Although rotaviruses can be found in the lamina propria and regional lymphatics, replication at these sites and systemic spread usually do not occur in immunocompetent persons (3).

Despite the superficial nature of mucosal infection, rotaviruses induce both local intestinal and systemic immune responses (17,18). Early animal studies suggested that the presence of rotavirus antibodies in the intestinal lumen (but not in the serum) was correlated with protection against disease. Oral administration of preparations containing rotavirus antibodies has successfully treated chronic rotavirus infection and diarrhea in immunocompromised children (19,20). In a randomized clinical trial, a single oral dose of gamma globulin reduced the duration of illness and the shedding of virus in infants hospitalized with rotavirus diarrhea. These observations indicate that intestinal immunity protects against rotavirus diarrhea and that the success of a rotavirus vaccine will depend, in part, upon its ability to induce mucosal immune responses.

In infants and young children, neutralizing antibodies directed primarily against the G serotype of the infecting strain (homotypic response) develop after primary infection with rotavirus (18). Repeat rotavirus infections elicit both a homotypic and heterotypic (against strains with different G serotypes) antibody response. Protection against rotavirus diarrhea correlates with serum antibody titers following natural infection of young children, and infected children are more protected against reinfection with similar rather than different G serotypes. A protective role of placentally transferred maternal antibody among infants <3 months of age has also been speculated since rotavirus disease is uncommon in this age group. However, serum neutralizing antibody responses among vaccine recipients have sometimes correlated poorly with protection from disease; therefore, the exact role of serum antibody in protection against disease remains unclear.

### Rotavirus Vaccines

### Monovalent "Jennerian" Vaccines

Initial development of rotavirus vaccines was based on the Jennerian approach, which involved the use of a live, attenuated, antigenically related virus derived from a nonhuman host (21). This approach was prompted by studies indicating that animal and human rotaviruses shared a common group antigen and that experimental animals immunized with animal strains of rotavirus had a significantly lower risk for illness and viral shedding when subsequently challenged with human rotaviruses. Furthermore, neutralizing antibodies to human rotavirus serotypes in the animal models indicated the potential for cross-protection.

### Bovine Vaccines

The first two Jennerian vaccines were developed with bovine rotavirus strains RIT4237 and WC3. The WC3 strain was passaged in cell culture less than RIT4237 and was developed because of concern that excessive passaging of the RIT vaccine might cause overattenuation and diminished efficacy. RIT4237 and WC3 were nonreactogenic and immunogenic when administered to infants 2 to 18 months of age. However, the protection conferred by both vaccines varied greatly in efficacy studies, 0% to 76% against any rotavirus diarrhea and 0% to 100% against severe disease (22-32). A well-defined correlate of protection was not identified, and reasons for the variable efficacy were unknown, although late age at vaccination, timing of vaccination with respect to the onset of the rotavirus season, and variations in the strength and number of doses of the vaccine were proposed as contributing factors. Both vaccines performed less well in developing than in industrialized countries, possibly because of interference by other enteropathogens or inadequate surveillance during follow-up.

### Rhesus Vaccine

The third Jennerian vaccine was developed with rhesus rotavirus strain MMU18006, which shares neutralization specificity with human rotavirus G3 strains. Besides sharing antigenic specificity with an epidemiologically important human rotavirus serotype, MMU18006 was suitable for vaccine development because it grew efficiently in cell culture. As in the bovine rotavirus-based vaccines, MMU18006 was safe and immunogenic, although in some trials, one third of infants became febrile 3 to 4 days after vaccination. The reactogenicity of MMU18006 was particularly high in two studies in Finland and Sweden in which 64% and 79% of infants, respectively, became febrile. Most children with febrile responses were >5 months of age; lack of passively transferred maternal antibody might have contributed to the high reactogenicity of the vaccine. As in the RIT4237 and WC3 vaccines, the protective efficacy of MMU18006 in field trials was quite variable, 0% to 60% against any rotavirus diarrhea and 0% to 85% against severe rotavirus diarrhea (33-39).

### Reassortant "Modified Jennerian" Vaccines

The greatest efficacy of MMU18006 was observed in a Venezuelan trial in which the rotavirus strain circulating in the community (G3) was the same serotype as the vaccine strain, which suggested that serotype-specific immunity against each of the epidemiologically important strains of human rotaviruses may be required for maximum protection. Similar observations in vaccine challenge cross-protection studies in animals initiated the development of vaccines that used a modified Jennerian approach in which animal-human reassortants expressing VP7 proteins of serotypes 1 through 4 were used as the immunogens.

### Rhesus-Human Reassortant Vaccines

Rhesus-human reassortants were generated by coinfecting cell cultures with rhesus rotavirus (RRV) strain MMU18006 (G serotype 3) and human rotavirus strains D (G serotype 1), DS-1 (G serotype 2), and ST3 (G serotype 4). Selection pressure (induced by the addition of neutralizing antibody to VP7 of RRV) produced reassortant strains D x RRV, DS-1 x RRV, and ST3 x RRV, each of which possessed the VP7 gene from HRV serotype 1, 2, or 4 and the other 10 genes from RRV (Figure 6) (40). Because vaccines made from the individual reassortants were safe and immunogenic, RRV-TV was developed incorporating each of the three reassortants and MMU18006 to provide coverage against the four common VP7 serotypes of rotavirus.

RRV-TV testing was initiated at an inoculum of $10^4$ PFU of each of the four viruses (i.e., at 4 x $10^4$ PFU) and completed at $10^5$ PFU, the dose submitted for licensure (i.e., at 4 x $10^5$ PFU). In most trials, vaccine was administered orally in three doses separated by at least 3 weeks to optimize the immune response to the component antigens; immunization was completed by the age of 6 to 7 months. Because the vaccine virus strains are acid labile, they are administered with 2.5 ml of citrate-bicarbonate buffer.

**Safety.** In clinical trials involving approximately 10,000 infants, the tetravalent vaccine has not been associated with any major adverse reactions. In a large multicenter trial in the United States, RRV-TV recipients had a small increase in febrile reactions (axillary temperature >38°C) on day 4 after the first dose (2.2% vs. 0.2%, $p = 0.02$) compared with placebo recipients;

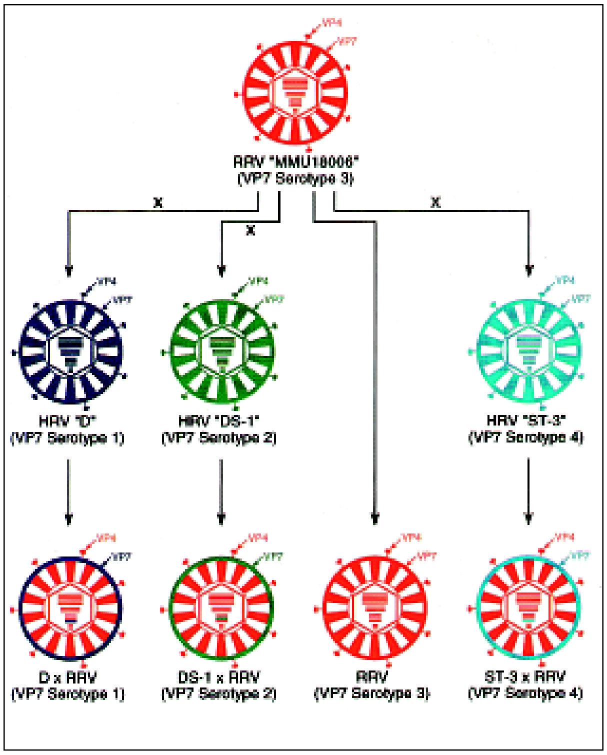

Figure 6. Schematic demonstration of production of rhesus rotavirus (RRV), human rotavirus (HRV) x rhesus rotavirus (RRV) reassortant quadrivalent vaccine with VP7 serotype 1, 2, 3, and 4 specificity. Reprinted with permission from (40).

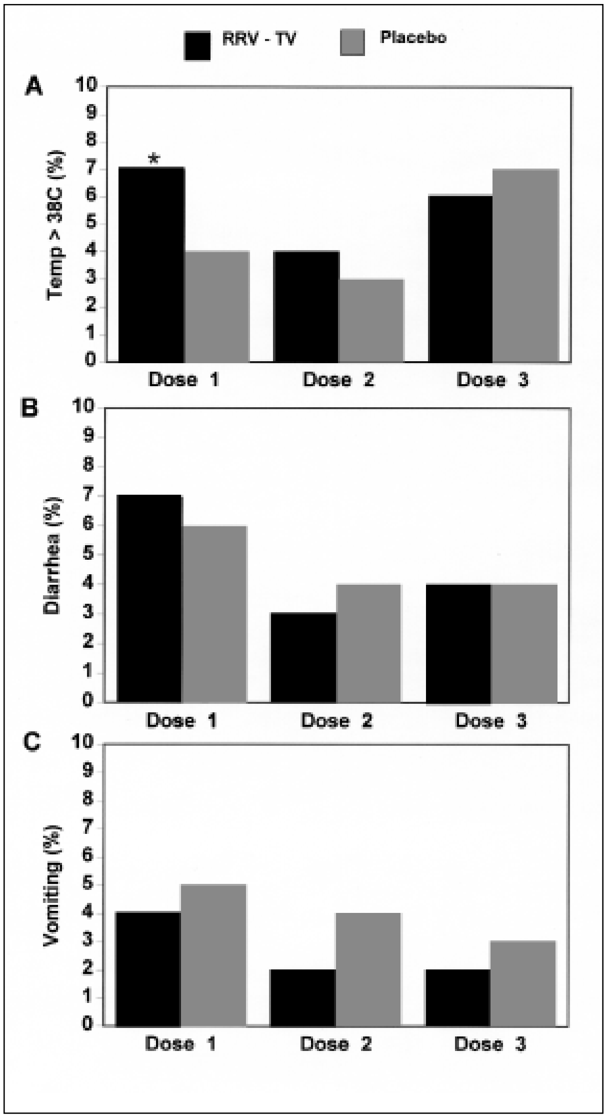

Figure 7. Percentage of children with adverse reactions during 5 days of surveillance following each of three doses of tetravalent rhesus-rotavirus vaccine (RRV-TV) or placebo. A) percentage with fever (as measured by axillary temperatures).*On day 4, 2.2% of RRV-TV recipients vs. 0.2% of placebo recipients became febrile (p = 0.02). B) percentage with diarrhea. C) percentage with vomiting. Adapted from (43).

no significant differences were observed in rates of fever after doses 2 and 3 and of vomiting and diarrhea after any dose (Figure 7). However, the fevers were mild compared with those associated with other childhood vaccines (e.g., diphtheria-tetanus-pertussis vaccine). The results of clinical trials of the monovalent parent RRV strain indicate that febrile reactions may be more frequent or severe among infants who receive the first dose of RRV-TV at >5 months of age.

In the Venezuelan trial of RRV-TV (41), PCR detected vaccine strains of rotavirus (in concentrations too low to be detected by routine methods such as EIA or polyacrylamide gel electrophoresis) in fecal samples from 15% of vaccinated and 13% of unvaccinated children. Although these data suggest the spread of vaccine virus to unvaccinated children, it is not known whether such spread would result in "herd protection" through induction of protective antibody responses in unvaccinated persons. Despite the theoretical risk for transmission of vaccine virus from RRV-TV recipients to household members and close contacts with known contraindications for receipt of RRV-TV (such as immunocompromised persons), this risk must be weighed against the benefit of protecting these immunocompromised persons by immunizing young children at risk for wild rotavirus infection.

**Efficacy.** Seven large efficacy trials have been completed using three doses of RRV-TV: four with the 4 X $10^5$ PFU dose submitted for licensure and three with a lower dose (4 X $10^4$ PFU) (Table) (41-47). The four trials at the 4 X $10^5$ PFU dose yielded similar results: the vaccine demonstrated significant protection against any rotavirus diarrhea (49% to 68%), greater protection against severe rotavirus diarrhea (61% to 91%), and 50% to 100% efficacy in preventing doctor visits for diarrhea. The efficacy of the vaccine in developing countries has been variable, but in a recent trial involving more than 2,200 underprivileged urban children in Venezuela, vaccine efficacy approached levels seen in industrialized countries (48% against all episodes of diarrhea, 70% against episodes requiring hospitalization, 75% against dehydrating illness, and 88% against severe episodes of rotavirus diarrhea) (41). This large trial is the first to clearly show the potential usefulness of rotavirus vaccines in developing countries, where they are most needed.

### Bovine-Human Reassortant Vaccines

Bovine-human reassortant rotavirus vaccines include a tetravalent WC3 rotavirus reassortant vaccine with genes coding for the VP7 of three major serotypes of rotavirus (G1, G2, and G3) and W179-4, a human VP4 reassortant with P[8] specificity. Theoretically, this vaccine should induce antibodies broadly reactive to the three common serotypes of rotavirus sharing P[8] specificity, thereby increasing the protective efficacy of this vaccine. In an efficacy trial of a three-dose regimen of the WC3 reassortant vaccine, protection was 67% against all rotavirus diarrhea and 69% against severe rotavirus diarrhea (48).

### Other Candidate Vaccines

In clinical trials, no Jennerian vaccine has provided complete protection against rotavirus diarrhea; as a result, several non-Jennerian candidate vaccines are being developed. Vaccines based on neonatal, cold-adapted, and attenuated human strains of rotavirus are under evaluation (49). Other approaches, such as the use of baculovirus-expressed viruslike particles or naked DNA vaccines, are also being used to develop candidate rotavirus vaccines (50,51).

### Implementing Rotavirus Vaccines

Introduction of rotavirus vaccines as routine childhood immunizations will be predicated on their safety, efficacy, expected impact, and cost. These aspects need to be addressed separately for industrialized and developing countries because of the difference in the epidemiology of rotavirus, the performance of rotavirus vaccines in clinical trials, and the logistics of implementing new vaccines.

In industrialized countries, the death rate from diarrheal illness has declined to a level that it no longer poses a serious threat to child survival. In the United States, diarrheal illnesses cause approximately 300 deaths per year, accounting for less than 2% of all childhood deaths. However, diarrhea still causes 170,000 hospitalizations each year, resulting in high medical and societal costs. Rotavirus is the most common pathogen identified in children hospitalized with diarrhea and is estimated to account for one third of all diarrheal illnesses. Large

Table. Efficacy of the tetravalent rhesus-human reassortant rotavirus vaccine (RRV-TV) in clinical trials

| Country (ref) | Age of vaccinees | No. enrolled vaccine/placebo | Vaccine dose (PFU) | No. doses | Circulating strains | Vaccine efficacy (%) All disease | Vaccine efficacy (%) Severe disease |
|---|---|---|---|---|---|---|---|
| Industrialized | | | | | | | |
| Finland (42) | 3-5 mo | 1,191/1,207 | 4 x $10^5$ | 3 | G1,G2 | 68 | 61-100 |
| United States (43) | 5-25 wk | 403/400 | 4 x $10^5$ | 3 | G1,G3 | 49 | 80-100 |
| United States (44) | 2-6 mo | 347/348 | 4 x $10^5$ | 3 | G3 | 50 | 64 |
| United States (45) | 4-26 wk | 332/330 | 4 x $10^4$ | 3 | G1,G3 | 57 | 82 |
| Developing | | | | | | | |
| Venezuela (41) | 8-18 wk | 1,112/1,095 | 4 x $10^5$ | 3 | G1 | 48 | 88 |
| Peru (46) | 2-4 mo | 219/209 | 4 x $10^4$ | 3 | G1,G2 | 24 | 0-40 |
| Brazil (47) | 1-5 mo | 233/233 | 4 x $10^4$ | 3 | G1,G2 | 35 | 46 |

multicenter trials in the United States have convincingly demonstrated that RRV-TV can safely prevent half of all mild rotavirus illnesses, 80% of severe episodes, and nearly all hospitalizations associated with rotavirus diarrhea. A routine, universal immunization program against rotavirus would prevent an estimated 227,000 physician visits, 95,000 emergency room visits, and 34,000 hospitalizations from diarrhea among children in the first 5 years of life (6). Therefore, even at a relatively high operational cost, an immunization program against rotavirus is likely to be cost-effective and can be recommended for the United States and probably for other industrialized countries on the basis of similar considerations.

In developing countries, recommending rotavirus vaccines for universal childhood immunization is more complex. With approximately 2,000 children dying each day from rotavirus diarrhea, the potential benefit of rotavirus vaccines is much greater in developing than in industrialized countries. Yet several questions remain unanswered.

Will rotavirus vaccines be as efficacious in developing countries as in industrialized countries? Several factors—younger age at infection, potentially larger inoculum of infection, presence of unusual strains of rotavirus, interference by other enteropathogens, and poorer nutritional status of children—could adversely affect the efficacy of rotavirus vaccines in developing countries. While the high efficacy observed in Venezuela is encouraging, further efficacy studies are needed in other parts of the developing world.

How many doses of vaccine are required for maximum protection against rotavirus diarrhea? In all clinical trials of the tetravalent rotavirus vaccine, three doses were administered to children 4 weeks to 6 months of age. For this reason and because of a lack of good correlation between neutralizing antibody response and protection from disease, it is difficult to determine whether fewer vaccine doses would confer the same level of protection. While fewer vaccine doses are appealing for programmatic and economic reasons, multiple vaccine doses may be required to overcome the factors that could reduce the efficacy in developing countries.

Finally, can the developing world afford another childhood vaccine? A vaccine priced in the \$10- to \$30-per-dose range may be cost-effective for industrialized countries but unaffordable for developing countries where the total per capita health-care expenditure may be \$10 to \$20 per year or less. Nonetheless, because the vaccine is prepared from virus grown in tissue culture, the price may be much lower once the developmental costs are recovered and the vaccine is produced and marketed locally. In addition, by recovering a major portion of manufacturing costs from the middle- and upper-class population, vaccine manufacturers may be able to provide vaccine to the underprivileged at a nominal price. Ultimately, the rate at which rotavirus vaccines are incorporated into immunization programs will depend not only on economic considerations but also on their perceived value by national governments and international and bilateral agencies (52).

### Acknowledgments

We thank the following for contributing illustrations: Dr. Charles Humphrey (Fig. 1), Dr. Mark Miller (Fig. 2), Drs. Mary Estes, Bidadi Prasad, and A. L. Shaw (Fig. 3), Dr. Thomas Török and Michael Bosley (Fig. 5 and the rotavirus map animations), and Dr. Albert Kapikian (Fig. 6). We thank Dr. Linda Han for helpful discussions and Anne Mather for editorial assistance.

Dr. Parashar is a medical epidemiologist with the Respiratory and Enteric Viruses Branch, DVRD, NCID, CDC. His research focuses on the epidemiology of viral gastroenteritis and methods for its prevention and control.

### References

1. Bishop RF, Davidson GP, Holmes IH, Ruck BJ. Virus particles in epithelial cells of duodenal mucosa from children with acute non-bacterial gastroenteritis. Lancet 1973;1:1281-3.
2. de Zoysa I, Feachem RV. Interventions for the control of diarrhoeal diseases among young children: rotavirus and cholera immunisation. Bull World Health Organ 1985;63:569-83.
3. Kapikian AZ, Chanock RM. Rotaviruses. In: Fields BN, Knipe DM, Howley PM, Chanock RM, Monath TP, Melnick JL, et al., editors. Fields virology, 3rd ed. Vol. 2. New York: Raven Press; 1996: p. 1657-708.
4. Estes MK. Rotaviruses and their replication. In: Fields BN, Knipe DM, Howley PM, Chanock RM, Monath TP, Melnick JL, et al, editors. Fields virology, 3rd ed. Vol. 2. New York: Raven Press; 1996. p. 1625-55.
5. Parashar UD, Holman RC, Clarke MJ, Bresee JS, Glass RI. Hospitalizations associated with rotavirus diarrhea in the United States, 1993 through 1995: surveillance based on the new ICD-9-CM rotavirus specific diagnostic code. J Infect Dis 1998;177:7-13.
6. Tucker AW, Haddix AC, Bresee JS, Holman RC, Parashar UD, Glass RI. Cost-effectiveness analysis of a rotavirus immunization program for the United States. JAMA 1998;279:1371-6.

7. Institute of Medicine. The prospects of immunizing against rotavirus. In: New vaccine development: diseases of importance in developing countries, Vol. 2. Washington: National Academy Press; 1986. p. D-13 to D-13-2.
8. Glass RI, Bresee JS, Parashar UD, Miller MA, Gentsch JR. Rotavirus vaccines at the threshold. Nat Med 1997;3:10-1.
9. Bishop RF, Barnes GL, Cipriani E, Lund JS. Clinical immunity after neonatal rotavirus infection: a prospective longitudinal study in young children. N Engl J Med 1983;309:72-6.
10. Velazquez FR, Matson DO, Calva JJ, Guerrero ML, Morrow AL, Carter-Campbell S, et al. Rotavirus infection in infants as protection against subsequent infections. N Engl J Med 1996;335:1022-8.
11. Gentsch JR, Woods PA, Ramachandran M, Das BK, Leite JP, Alfieri A, et al. Review of G and P typing results from a global collection of rotavirus strains: implication for vaccine development. J Infect Dis 1996;174:S30-6.
12. Ramachandran M, Das BK, Vij A, Kumar R, Bhambal SS, Kesari N, et al. Unusual diversity of human rotavirus G and P genotypes in India. J Clin Microbiol 1996;34:436-9.
13. Cicirello HG, Das BK, Gupta A, Bhan MK, Gentsch JR, Kumar R, et al. High prevalence or rotavirus infection among neonates born at hospitals in Delhi, India: predisposition of newborns for infection with unusual rotavirus. Ped Infect Dis J 1994;13:720-4.
14. Cook SM, Glass RI, LeBaron CW, Ho M-S. Global seasonality of rotavirus infections. Bull World Health Organ 1990;68:171-7.
15. Török TJ, Kilgore PE, Clarke MJ, Holman RC, Bresee JS, Glass RI. Visualizing geographic and temporal trends in rotavirus activity in the United States, 1991 to 1996. Pediatr Infect Dis J 1997;16:941-6.
16. Nakagomi O, Nakagomi T. Genetic diversity and similarity among mammalian rotaviruses in relation to interspecies transmission of rotavirus. Arch Virol 1991;120:43-55.
17. Ward R. Mechanisms of protection against rotavirus in humans and mice. J Infect Dis 1996;174:S51-8.
18. Offit PA. Host factors associated with protection against rotavirus disease: the skies are clearing. J Infect Dis 1996;174:S59-64.
19. Ebina T, Sato A, Umezu K, Ishida N, Ohyama S, Ohizumi A, et al. Prevention of rotavirus infection with cow colostrum containing antibody against human rotavirus. Lancet 1983;2:1029-30.
20. Hilpert H, Brussow H, Mietens C, Sidoti J, Lerner L, Werchau H. Use of bovine milk concentrate antibody to rotavirus to treat rotavirus gastroenteritis in infants. J Infect Dis 1987;156:158-66.
21. Kapikian AZ. Jennerian and modified Jennerian approach to vaccination against rotavirus diarrhea in infants and young children: an introduction. In: Kapikian AZ, editor. Viral infections of the gastrointestinal tract. Vol 2. New York: Marcel Dekker; 1994. p. 409-17.
22. Vesikari T, Isolauri E, d'Hondt E. Protection of infants against rotavirus diarrhea by RIT 4237 attenuated bovine rotavirus strain vaccine. Lancet 1984;1:977-81.
23. Vesikari T, Isolauri E, Delem A, d'Hondt E, Andre FE, Beards GM, et al. Clinical efficacy of the RIT 4237 live attenuated bovine rotavirus vaccine in infants vaccinated before a rotavirus epidemic. J Pediatr 1985;107:189-94.
24. Vesikari T, Ruuska T, Delem A, Andre FE, Beards GM, Flewett TH. Efficacy of two doses of RIT 4237 bovine rotavirus vaccine (at birth and 7 months of age) for prevention of rotavirus diarrhea. Acta Paediatrica Scandinavica 1991;80:173-80.
25. Ruuska T, Vesikari T, Delem A, Andre FE, Beards GM, Flewett TH. Evaluation of RIT 4237 bovine rotavirus vaccine in newborn infants: correlation of vaccine efficacy and season of birth in relation to rotavirus epidemic period. Scand J Infect Dis 1990;22:269-78.
26. Santosham M, Letson WG, Wolff M, Reid R, Gahagan S, Adams R, et al. A field study of the safety and efficacy of two candidate rotavirus vaccines in a Native American population. J Infect Dis 1991;163:483-7.
27. Hanlon P, Hanlon L, Marsh V, Byass P, Shenton F, Hassan-King M, et al. Trial of an attenuated bovine rotavirus vaccine (RIT 4237) in Gambian infants. Lancet 1987;1:1342-5.
28. DeMol P, Zissis G, Butzler JP, Mutwewingabo A, Andre FE. Failure of live, attenuated oral rotavirus vaccine. Lancet 1986;1:108.
29. Lanata CF, Black RE, del Aguila R, Gil A, Verastegui H, Gerna G, et al. Protection of Peruvian children against rotavirus diarrhea of specific serotypes by one, two, or three doses of the RIT 4237 attenuated bovine rotavirus vaccine. J Infect Dis 1989;159:452-9.
30. Clark HF, Borian FE, Bell LM, Plotkin SA. Protective effect of WC3 vaccine against rotavirus diarrhea in infants during a predominantly serotype 1 rotavirus season. J Infect Dis 1988;158:570-87.
31. Bernstein DI, Smith VE, Sander DS, Pax KA, Schiff GM, Ward RL. Evaluation of WC3 rotavirus vaccine and correlates of protection in healthy infants. J Infect Dis 1990;162:1055-62.
32. Georges-Courbot MC, Monges J, Siopathis MR, Roungou JB, Gresenguet G, Bellec L, et al. Evaluation of the efficacy of a low-passage bovine rotavirus (strain WC3) vaccine in children in Central Africa. Res Virol 1991;142:405-11.
33. Rennels MB, Losonsky GA, Levine MM, Kapikian AZ. Preliminary evaluation of the efficacy of rhesus rotavirus vaccine strain MMU 18006 in young children. Pediatr Infect Dis J 1986;5:587-8.
34. Gothefors L, Wadell G, Juto P, Taniguchi K, Kapikian AZ, Glass RI. Prolonged efficacy of rhesus rotavirus vaccine in Swedish children. J Infect Dis 1989;159:753-7.
35. Christy C, Madore HP, Pichichero ME, Gala C, Pincus P, Vosefsky D, et al. Field trial of rhesus rotavirus vaccine in infants. Pediatr Infect Dis J 1988;7:645-50.
36. Rennels MB, Losonsky GA, Young AE, Shindledecker CL, Kapikian AZ, Levine MM. An efficacy trial of the rhesus rotavirus vaccine in Maryland. The clinical study group. Am J Dis Child 1990;144:601-4.
37. Vesikari T, Rautenen T, Varis T, Beards GM, Kapikian AZ. Rhesus rotavirus candidate vaccine. Am J Dis Child 1990;144:285-9.

38. Madore HP, Christy C, Pichichero M, Long C, Pincus P, Vosefsky D, et al. Field trial of rhesus rotavirus or human-rhesus reassortant vaccine of VP7 serotype 3 or 1 specificity in infants. J Infect Dis 1992;166:235-43.
39. Perez-Schael I, Garcia D, Gonzalez M, Gonzalez R, Daoud N, Perez M, et al. Prospective study of diarrheal diseases in Venezuelan children to evaluate the efficacy of rhesus rotavirus vaccine. J Med Virol 1990;30:219-29.
40. Kapikian AZ, Hoshino Y, Chanock RM, Perez-Schael I. Efficacy of a quadrivalent rhesus rotavirus-based human rotavirus vaccine aimed at preventing severe rotavirus diarrhea in infants and young children. J Infect Dis 1996;174:S65-72.
41. Perez-Schael I, Guntinas MJ, Perez M, Pagone V, Rojas AM, Gonzlez R, et al. Efficacy of the rhesus rotavirus-based quadrivalent vaccine in infants and young children in Venezuela. N Engl J Med 1997;337:1181-7.
42. Joensuu J, Koskenniemi E, Pang X-L, Vesikari T. A randomized, double-blind placebo controlled trial of rhesus-human reassortant rotavirus vaccine for prevention of severe rotavirus gastroenteritis. Lancet 1997;350:1205-9.
43. Rennels MB, Glass RI, Dennehy PH, Bernstein DI, Pichichero ME, Zito PT, et al. Safety and efficacy of high-dose rhesus-human reassortant rotavirus vaccines-report of the national multicenter trial. Pediatrics 1996;97:7-13.
44. Santosham M, Moulton LH, Reid R, Croll J, Weatherholt R, Ward R, et al. Efficacy and safety of high-dose rhesus-human reassortant rotavirus vaccine in Native American populations. J Pediatr 1997;131:632-8.
45. Bernstein DI, Glass RI, Rodgers G, Davidson BL, Sack DA, U.S. Rotavirus Vaccine Efficacy Group. Evaluation of rhesus rotavirus monovalent and tetravalent reassortant vaccines in U.S. children. JAMA 1995;273:1191-6.
46. Lanata CF, Midthun K, Black RE, Lazo F, Butron B, Linares A, et al. Safety, immunogenicity, and protective efficacy of one and three doses of the tetravalent rhesus rotavirus vaccine in infants in Lima, Peru. J Infect Dis 1996;174:268-75.
47. Linhares AC, Gabbay YB, Mascarenhas JDP, deFreitas RB, Oliveira CS, Bellesi N, et al. Immunogenicity, safety, and efficacy of tetravalent rhesus-human, reassortant rotavirus vaccine in Belem, Brazil. Bull World Health Organ 1996;74:491-500.
48. Clark HF, Offit PA, Ellis RW, Eiden JJ, Krah D, Shaw AR, et al. The development of multivalent bovine rotavirus (strain WC3) reassortant vaccine for infants. J Infect Dis 1996;174:S73-80.
49. Vesikari T, Ruuska T, Koivu H, Green KY, Flores J, Kapikian AZ. Evaluation of the M37 human rotavirus vaccine in 2- to 6-month-old infants. Pediatr Infect Dis J 1991;10:912-7.
50. Connor ME, Zarley CD, Hu B, Parsons S, Drabinski D, Greiner S, et al. Virus-like particles as a rotavirus subunit vaccine. J Infect Dis 1996;174:S88-92.
51. Herrmann JE, Chen SC, Fynan EF, Santoro JC, Greenberg HB, Wang S, et al. Protection against rotavirus infections by DNA vaccination. J Infect Dis 1996;174:S93-7.
52. Hausdorff WP. Prospects for the use of new vaccines in developing countries: cost is not the only impediment. Vaccine 1996;14:1179-86.
